# The Effect of Modified TURP (M-TURP) in Intra and Postoperative Complications

**DOI:** 10.5812/numonthly.6607

**Published:** 2013-03-30

**Authors:** Ali Asghar Ketabchi, Mahsa Ketabchi, Mohsen Barkam

**Affiliations:** 1 Urology Department, Physiology Research center, Kerman Medical Sciences University, Kerman, IR Iran

**Keywords:** Transurethral Resection of Prostate, Intraoperative Complications, Postoperative Complications

## Abstract

**Background:**

Transurethral resection of the prostate (TURP) is the most common surgical treatment for benign prostatic hyperplasia.

**Objectives:**

The aim of the present study was to compare the conventional bipolar transurethral resection of prostate (TURP) with a modified transurethral resection of the prostate (M-TURP) in men with mild to moderate symptoms of benign prostatic hyperplasia.

**Patients and Methods:**

To compare and evaluate the clinical outcomes of M-TURP, a new electrosurgical suggested method, with the standard treatment, transurethral resection of prostate (TURP), 200 patients with benign prostatic enlargement causing moderate to severe clinical lower urinary symptoms were selected and divided into two equal groups of A and B. Patients of group A underwent M-TURP (incomplete bladder neck resection), resecting only from 1 to 11 O'clock position and group B underwent conventional TURP. These patients were evaluated between Jun 2008 and April 2011, after excluding 24 patients, finally 176 men were studied, 98 in the conventional monopolar transurethral resection of prostate (TURP) group and 78 in the (M-TURP) group. Postoperative follow up to assess the results of the surgeries and the complication rates, began from the operation and continued with postoperative visits of the patient at 24 hour after the catheter remove, two weeks, three months and finally six months.

**Results:**

The age range of both groups were the same (65-82 years old), preoperative IPSS score in study and control groups were 18 ± 3.3, 17 ± 4.6 (nonsignificant P value = ns) respectively. The size of prostate gland was 58 ± 3.5 g in study and 78 ± 1.2 g in control (ns) preoperatively. Intra and postoperative complications including hematuria (need for transfusion), urine retention (need for catheterization), fever after operation in study and control groups were 2.04%, 6.41%, 1.02% and 0.0%, 3.06%, 6.41% respectively. ISI score (stress incontinence score index) were 7 ± 2.5 and 19 ± 3.6 and UR (urge ratio) were %26 and %70 for study and control groups respectively; P < 0.05. IIEF (international index of erectile function) in study group was better than control (23 ± 3.2 vs. 11 ± 1.7), P < 0.05.

**Conclusions:**

The results of this study showed that the support of anterior fibro muscular zone (anterior lobe) of prostate after TUR-P has a significant role in postoperative complications, especially in postoperative stress incontinence. So, we strongly recommend to preserve this segment of prostate for prevention of incontinence and other intra and postoperative complications.

## 1. Background

The aetiology of BPH is not completely understood, but it seems to be multifactorial and endocrine controlled. Androgens (testosterone and related hormones) are considered to play a permissive role in BPH by most experts. DHT is produced from testosterone by 5α-reductase type 2 in prostate gland. The both stromal and epithelial elements of prostate can give rise to hyperplastic tissues and cause BPH related symptoms.

BPH is the fourth diagnosis in men over 50 years old (1). BPH ranks about the seventh for costs, when looking at one-year disease-specific medical costs in men over 50 years old (2). Half of all men have BPH identifiable histological at the age of 60, and by 85, the prevalence is about 90%. In the USA about 25% of men would be treated for BPH by age 80 years old.

The main aim of LUTs treatment related to BPH should relieve symptoms and qualify the life style of these patients and also prevent progression of disease and the development of complications. The beneficial effects need to be balanced against the potential side effects of treatment.

Indications for surgery have changed widely over the time, and in the current era they are much more conservative than 10 to 20 years ago. Certain absolute or near absolute indications exist like refractory or repeated urinary retention that 12.5 percent of patients with BPH had experienced an acute urinary retention (AUR) event, from them 7.2 percent had undergone prostate surgery. The average cost of an AUR event was $369 and surgery was $5,699 (3).

The other Indications for surgical prostatectomy are obstructive uropathy due to BPH, recurrent gross hematuria, recurrent or persistent UTI due to BPH, Bladder stone, significant residual volume, overflow incontinence, and large bladder diverticula due to BPH.

Without a main indication, or combinations of those aforementioned, the bothersome nature of the symptoms and low quality of life is usually what compels the patient to search for treatment, or the physician to suggest treatment. Abnormal urodynamic results may also have a role as well.

More than 300,000 prostatectomies are performed each year (mostly transurethral resection of the prostate, TURP). After cataract surgery the TURP is the second most common surgical procedure, at a cost about $2 billion per year.

Transurethral resection of prostate (TURP) is currently the gold standard for surgical treatment of benign prostatic hyperplasia (BPH), as this procedure results in the best improvement in symptoms and urine flow rate (4). Nevertheless, the TURP have some complications. Mebust et al. reported an 18% morbidity rate after TURP and a meta-analysis by the BPH guideline panel showed that the morbidity rate associated with TURP ranges from 7% to 43% (5). This has led to the creation of new alternative methods of treatment for BPH for reducing complications, morbidity, hospital stay and cost.

We have seen major changes and developments in the TURP technique in the last decade which has had great impact on the incidence of intra- and postoperative complications. According to the European society of urotechnology, we concentrated on actual TURP practices, to qualify and update the status, technical advancement of TURP, prevention, and management of complications (6).

Recent TURP technique developments in Germany by Mauermayer (7), Hartung and May (8) have gained popularity. TURP is traditionally divided into four steps: midlobe resection, paracollicular resection, resection of lateral lobes and ventral parts, and apical resection. From these recent developments are suprapubic trocar systems (9) and continuous-flow resectoscopes (10), which both have improved irrigation pressure. Another main development in this field was video-assisted resection (11).

Despite these improvements in surgical techniques and specialized anesthesiology, a perioperative mortality rate of 0.2% and a delayed mortality due to cardiovascular diseases are still a significant risk factor (12).

## 2. Objectives

The aim of the present study was to compare the conventional bipolar transurethral resection of prostate (TURP) with a modified transurethral resection of the prostate (M-TURP) in men with mild to moderate symptoms of benign prostatic hyperplasia.

## 3. Patients and Methods

The study was conducted at the Department of Urology, hospital Bahonar Kerman, Iran over a period of Three years (2008 - 2011). A total of 200 patients, with mild to moderate lower urinary tract symptoms due to benign prostatic hyperplasia were recruited. Informed consent was given from each patient. They were randomised blindly into two groups of one hundred patients. One group (A) underwent M-TURP, while the other one (B) underwent the standard TURP.

Patients with bleeding disorders, diabetes mellitus, cardiac failure, neurological disorders, renal function impairment, liver disorders, vesical calculi, urethral strictures, carcinoma prostate or prostate larger than 100 gm were excluded from the study (n = 24).

A detailed history was taken and a thorough examination was performed. It provided necessary information about patients’ symptoms and their condition. All patients had urine analysis, and urine cultures were also performed in case of any sing of infection. Complete blood count, electrolytes, creatinine, urea, sugar, clotting and bleeding time, and postoperative serum sodium were measured. Also ultrasonography, plain X-Ray (K.U.B = kidney. ureter .bladder), and ECG (electrocardiography) were performed.

Spinal or epidural anaesthesia was used. Preoperatively at the time of induction, prophylactic antibiotic was administered. During the TURP, the standard technique was followed using 24Fr size resectoscope (Karl Storz) with cutting loop and 30 degrees telescope. The resection was performed till the prostatic capsule, in whole circular from 12 0’clock to 12 0’clock, through the bladder neck till the verumontanum. Except in M-TURP procedure we resected the prostate tissue from 1 0’clock to 11 0’clock (with preserving the anterior segment "11 to 1").

In both procedures we used 1.5% glycine for irrigation purpose. Peri- operatively, pulse and B.P (blood pressure) were recorded every 15 minutes. Development of any other symptoms during the operation was also noted. 22 Fr three-way Foley’s urethral catheter was inserted after the operation, 0.9% saline was used for postoperative bladder irrigation. In group A, the urethral catheter was removed after 24 hours, while group B had their catheters removed after 72 hours.

Twenty four hours after removal of the catheter, symptom scoring was performed and note made of any complication, if present in this period. At this stage, the patients were discharged with instructions to visit the department at 2 weeks, 3 months and finally 6 months. Symptom scoring (international prostatic symptom score/IPSS), post micturating residual volume, uroflowmetry, urine examination, bacterial count and assessment for late complications were performed on these visits. Urethrogram was performed if there was any indication to exclude iatrogenic urethral strictures. Also between 3 to 6 months, all patients of both groups completed IIEF and QOL questionnaires and performed the uroflowmetry test (Q.max). Comparison of the two modalities was performed regarding their safety, efficacy and cost effectiveness (Table 1).

**Table 1.** Peri and Postoperative Findings Comparison of Both Groups

Variables	Group A (M-TURP)	Group B (TURP)	P value
**Hospital stay, h**	24.14 ± 7.86	48.2 ± 7.47	< 0.05
**Incontinence (ISI-score)**	7 ± 2.5	19 ± 3.6	< 0.05
**Hemorrhage**	1 ± 1.2	5.2 ± 2.2	< 0.01
**Infection (UTI)**	12 (%15.36)	17 (%17.34)	0.454
**IPSS-score**	5 ± 3.3	4 ± 2.7	0.622
**QOL**	2 ± 1.5	3 ± 2.7	0.552
**PVR, mL**	98 ± 4.52	120 ± 3.12	0.212
**Q.max, mL**	10 ± 1.7	11 ± 2.2	0.118
**Hb, g/dL**	13.35 ± 1.80	11.52 ± 2.00	0.14
**IIEF**	18 ± 3.2	10 ± 1.7	0.05

## 4. Results

The age range of both groups were the same (65-82 years old), preoperative IPSS score in study and control groups were 18 ± 3.3, 17 ± 4.6 (nonsignificant P value = ns) respectively ,the size of prostate gland preoperatively were 58 ± 3.5 g in study and 78 ± 1.2 g in control (ns), complications as hematuria (need for transfusion), urine retention (need for catheterization), fever after the operation (need for hospitalization) were 0.5% and 0.75%, 0.25% and 0.0% in study and control groups respectively. Incontinence ISI-score (stress incontinence score index) were 7 ± 2.5 and 19 ± 3.6 and UR (Urge Ratio) were %26 and %70 after the operation for study and control groups respectively, P < 0.05 , P < 0.05; IIEF (International Index of Erectile Function) in study group was better than control (11 ± 1.7 *vs.* 18 ± 3.2), P < 0.05 (Table 1 and 2).

**Table 2.** Preoperative Data of Both Groups

Variables	Group A (M-TURP)	Group B (TURP)
**Mean Age, y**	64.14 ± 7.86	67.2 ± 7.47
**Prostate Mean Weight, g**	58 ± 3.5	78 ± 1.2
**Pre-op Serum PSA, ng/mL**	6.1 ± 1.2	5.2 ± 2.2
**Pre-op Serum Sodium, mEq/L**	139.8 ± 3.50	140.15 ± 2.3
**IPSS-score**	18 ± 3.3	17 ± 4.6
**QOL**	4.5 ± 1.2	4.45 ± 0.87
**PVR, mL**	98 ± 4.52	120 ± 3.12
**Qmax, mL**	4.8 ± 1.7	5.2 ± 2.5
**Hb, g/dL**	12.35 ± 1.80	12.52 ± 2.00

## 5. Discussion

The popularity of TURP was cultivated by reported symptomatic and functional improvements repeatedly documented in clinical trials. Although, transurethral resection of the prostate (TURP) has long been regarded as the gold standard treatment for benign prostatic hyperplasia (BPH). However, TURP has also been associated with well-documented, significant peri- and postoperative risks (1-3). However TURP remains a standard method versus other methods of BPH treatment.

Since to date many other treatments have been offered as alternatives to TURP. Most have not approached TURP with respect to durability or efficacy, although morbidity is often improved. In men with larger prostates, the alternatives are even more limited. The characteristics of the M-TURP determine its versatility and provide an endoscopic alternative to both regularly TURP and open prostatectomy when used for enucleation.

In our study, M-TURP procedure by leaving the anterior segment of prostate was superior regarding perioperative morbidity, with reduced bladder irrigation and catheter times and reduced hospital stay.

Better quality of life in this study may be related to have fewer complications than controls, although this difference is not significant and this may be due to our small samples. As previous studies have shown this type of complications cause depression and reduce quality of life.

The goal of this study was to present M-TURP procedure benefits compared to TURP, so according to our findings in current study M-TURP procedure can be a valid alternative in medium size BPH by leaving the anterior segment of prostate (< 100g), while other advanced methods as laser techniques in medium-sized prostate have no advantages over TURP (13-15). Though there is potentially no limit to the size of a prostate that can be treated with laser techniques as Holmium laser enucleation of the prostate (HOLEP) (16).

The results of this study showed that the support of anterior fibro muscular zone (anterior lobe) of prostate after TUR-P has significant role in postoperative complications, especially in postoperative stress incontinence. So, we strongly recommend to preserve this segment of prostate for prevention of incontinence and other intra and postoperative complications.
